# Levels of autistic traits in anorexia nervosa: a comparative psychometric study

**DOI:** 10.1186/1471-244X-13-222

**Published:** 2013-09-10

**Authors:** Annaig Courty, Anne Solène Maria, Christophe Lalanne, Damien Ringuenet, Christine Vindreau, Coralie Chevallier, Lydia Pouga, François Pinabel, Anne Philippe, Jean-Louis Adrien, Caroline Barry, Sylvie Berthoz

**Affiliations:** 1 Department of Adolescent and Young Adult Psychiatry, Service de Psychiatrie Institut Mutualiste Montsouris, 42 Bd Jourdan, Paris 75014, France; 2 LPPS - EA 4057, Institut de Psychologie, Paris Descartes University, Paris, France; 3 Department of Psychiatry and Addictology, Eating disorders unit, AP-HP, Hôpital Paul Brousse, Paris Sud University, Villejuif, France; 4 Inserm U960 – LNC, Paris, France; 5 Center for Autism Research, Children’s Hospital of Philadelphia, Philadelphia, PA, USA; 6 Inserm U669 – Maison de Solenn, Paris Descartes and Paris Sud Universities, Paris, France; 7 Department of Child and Adolescent Psychiatry, AP-HP, Hôpital Pitié-Salpétrière, Paris Descartes University, Paris, France; 8 Private Practice, 6 avenue de Tourville, Paris 75007, France; 9 Inserm U781 & Department of Genetic, AP-HP, Hôpital Necker-Enfants Malades, Paris Descartes University, Paris, France; 10 AP-HP, Department of Clinical Research, Saint-Louis Hospital, Paris, France; 11 Inserm Unit UMR-SO 669, University Paris Sud, Paris Descartes, Paris, France

**Keywords:** Anorexia nervosa, Autism, Neuropsychology, Flexibility, Interpersonal functioning, Empathy, Alexithymia

## Abstract

**Background:**

A number of characteristics associated with Autism Spectrum Disorders (ASD) are over-represented among patients with Anorexia Nervosa (AN) as well as among relatives of these patients. Yet the co-occurrence of autistic traits in AN has not been fully explored and no previous study has directly compared self-reported evaluations of cognitive and socio-affective skills in AN and ASD.

**Methods:**

We aimed to determine the degree of overlap between AN and ASD from scores on questionnaires classically used to measure ASD impairments. Fifteen AN participants, 15 ASD participants and two groups of matched controls completed a battery of self-reports measuring: autistic traits (Autism-Spectrum Quotient), empathy (Empathy Quotient-short and Interpersonal Reactivity Index), systemizing (Systemizing Quotient-short) and alexithymia (Bermond-Vorst Alexithymia Questionnaire-B). Univariate comparisons of mean totalled scores were performed on each measure (patients vs. controls, and AN vs. ASD), and a Principal Component Analysis was used to study subject proximities in a reduced-factor space constructed from AQ, BVAQ-B and IRI subscales.

**Results:**

These analyses revealed similarities in a few cognitive domains (Attention Switching, Perspective Taking and Fantasy, lack of emotional introspection) and in some nonspecific affective dimensions (depression and feelings of distress), but also marked dissimilarities in social skills (the ability to communicate emotions to others, empathizing).

**Conclusion:**

The AN and ASD participants reported similar needs for sameness, and similar difficulties understanding their emotions and taking the perspective of another, but contrasting abilities to feel concerned in interpersonal situations. Our mixed findings encourage further exploration of transdiagnostic similarities and associations between these disorders.

## Background

There is a growing consensus that a better characterization of social and cognitive impairments in anorexia nervosa (AN), which overlap with those found in Autism Spectrum Disorders (ASD), may help to develop better approaches tailored to enhance specific areas of social functioning [1,2].

From a clinical perspective, a well-known Swedish prospective study on teenage-onset AN suggests that autistic symptomatology is over-represented in AN, that it is associated with poor outcome, and persists long-term after recovery [3,4]. However, using well validated clinical assessment tools to assess autistic symptoms, a more recent study comparing children and adolescents with an early-onset eating disorder (EOED), others with ASD and typically developing controls, failed to find an increased prevalence of ASD among the EOED, but reported clinically significant levels of autistic traits (in particular repetitive and stereotyped behaviours) [5]. Regarding cognitive functioning, AN patients, often display lack of flexibility (poor set-shifting) and extreme attention to details (weak central coherence), two features commonly found in ASD. Interestingly, in AN patients, these cognitive difficulties, extend beyond the sole effects of starvation, and are often shared with family members [6-10].

While atypical emotional and interpersonal functioning of individuals with ASD has been extensively studied, it is an emerging area of research in AN. Several recent studies showed that AN patients have higher scores on the Autism-spectrum Quotient (AQ) than healthy controls [4,11] and that they have difficulties processing their own as well as others’ emotions [11-18]. Yet the co-existence of autistic traits in AN is still little explored and the question of whether inflexibility and impairments in social cognitions in AN are ‘truly autistic’ remains unanswered [5], p.589. In particular, no previous study has directly compared self-reported social cognitive skills in AN and ASD adults.

Here, we report findings collected as part of an ongoing study comparing AN and ASD socio-affective functioning (DETENDOEMO; RGB:2007-A01068-45). As recommended [5], we used a dimensional approach to explore the extent to which AN and ASD patients present dissimilar scores on questionnaires classically used to measure ASD impairments. In addition, the level of symptoms and concerns characteristic of eating disorders and the level of current dysphoric affects were measured. We expected to replicate previous studies [4,11,17] suggesting that, relative to healthy controls, patients with AN have higher scores on questionnaires measuring autism-spectrum traits and alexithymia. Moreover, given the aforementioned socio-cognitive difficulties observed in AN, we predicted that the AN group would have lower scores on the empathy quotient and higher scores on the systemizing quotient questionnaires than their control group (despite previous negative results [11]). Conversely, relative to the ASD group, we expected the AN group to have similar scores on the autism-spectrum quotient and the alexithymia questionnaires, but higher scores on the empathy quotient and lower scores on the systemizing quotient questionnaires.

## Methods

All participants gave their written informed consent. The study was approved by the Ethics Committee, Paris Ile de France VI.

### Participants

A group of 15 adults with AN (5 restrictive and 10 binge/purging AN) was age-matched to a group of ASD participants (Table 1). Participants with AN (14 female, 1 male) had received a community diagnosis based on DSM-IV-TR criteria, which was confirmed using the Mini-Neuropsychiatric Interview (MINI, [19]). They were included during the second part of their hospital stay in a specialised inpatient unit (Hôpital P. Brousse). Expert clinician judgment (AC, CV, DR) was used to rule out a comorbid diagnosis of Autism or Pervasive Developmental Disorder. Mean (standard deviation) illness onset was 19.8(3.4) years and mean illness duration was 4.0(3.5) years.

**Table 1:** Descriptive and between-group statistics for each test measures (all complete cases)

	**ASD**	**ASD-C**	**AN**	**AN-C**
**Age**	28.1 (7.5) [19–43]	28.1 (7.3) [18–41]	23.9 (4.7) [19–37]	24.0 (4.9) [18–36]
**BMI**	23.2 (5.0) [17–35]	22.2 (3.0) [20–32]	16.4 (1.7) [12–19]	21.0 (1.8) [18–25]
**BDI-13**	5.2 (4.3) [0–15]	3.3 (3.7) [0–13]	8.5 (8.3) [0–31]	4.6 (4.8) [0–14]
**EAT Total**	5.3 (5.0) [0–13]	3.9 (5.1) [0–18]	16.9 (11.0) [5–43]	6.5 (4.7) [0–15]
***EAT Dieting***	3.9 (8.9) [0–11]	2.9 (3.9) [0–13]	8.9 (5.6) [2–21]	5.3 (3.8) [0–13]
***EAT Bulimia***	0.7 (1.2) [0–3]	0.4 (1.1) [0–4]	3.4 (4.7) [0–14]	0.7 (1.1) [0–3]
***EAT Oral***	0.7 (2.1) [0–8]	0.5 (0.9) [0–3]	4.5 (4.7) [0–18]	0.4 (0.6) [0–2]
**AQ Total**	33.1 (7.3) [22–45]	13.4 (5.1) [7–23]	20.3 (5.9) [11–31]	14.8 (4.9) [9–21]
***AQ Social***	7.4 (2.2) [4–10]	1.9(1.2) [0–4]	3.1 (2.3) [0–8]	2.1 (1.3) [0–5]
***AQ Switching***	7.9 (1.9) [5–10]	3.5(1.9) [1–8]	5.6 (2.2) [2–9]	3.0 (1.7) [0–5]
***AQ Details***	5.7 (2.6) [0–9]	3.7(2.6) [0–8]	5.6 (1.4) [3–8]	4.7 (2.2) [2–8]
***AQ Communication***	6.6 (2.0) [4–10]	1.6(1.5) [0–5]	2.7 (1.9) [1–6]	1.7 (1.8) [0–6]
***AQ Imagination***	5.5 (1.9) [3–10]	2.7(1.4) [0–5]	3.4 (2.0) [1–8]	3.3 (1.3) [1–5]
**EQ-short**	10.1 (5.7) [2–18]	19.9(3.4) [16–27]	23.0 (6.8) [8–31]	21.1 (7.4) [9–38]
**SQ-short**	23.0 (11.5) [5–43]	21.6(7.3) [8–34]	15.7 (6.1) [7–24]	16.5 (7.9) [5–30]
**IRI PT**	14.8 (3.8) [10–23]	17.1 (2.7) [13–23]	17.0 (4.9) [7–22]	17.2 (3.5) [11–25]
**IRI FS**	17.8 (5.5) [3–26]	17.6 (2.8) [14–22]	17.1 (4.8) [9–26]	21.1 (3.1) [17–26]
**IRI EC**	15.0 (5.1) [3–21]	19.0 (2.8) [16–25]	21.5 (4.7) [9–27]	20.9 (3.8) [15–27]
**IRI PD**	17.9 (4.5) [10–25]	13.3 (4.8) [6–25]	15.2 (4.9) [7–24]	14.7 (4.6) [6–23]
**BVAQ-B Total**	54.9 (10.7) [37–76]	46.6 (5.4) [36–53]	49.7 (11.9) [34–72]	41.2 (5.7) [31–53]
***BCOG***	35.7 (8.4) [25–53]	27.6 (4.3) [21–36]	30.6 (9.1) [18–48]	25.2 (4.9) [17–32]
***BAFF***	19.3 (5.1) [10–29]	19.0 (2.8) [15–25]	19.1 (4.1) [11–24]	16.0 (3.2) [10–21]

Data in cells represent Mean (SD) and [min-max].

ASD participants were addressed to our research group by expert clinicians (FP, AP) after having received a formal diagnosis of an Autism Spectrum Disorder according to DSM-IV-TR criteria. In addition, current ASD presentation was assessed using the Autism Diagnostic Observational Schedule (ADOS, [20]), M(SD) = 10.7 (2.4)). The MINI was used to rule out current eating disorder in the ASD group and form D of the French short version of the WAIS-III [21] was used to rule out intellectual disabilities. The ASD participants mean (standard deviation) IQ score was 109.5 (18.6). In this group, weight and height were self-reported, but participants were informed that this information was important for a subsequent neuroimaging study.

The ASD group and the AN group were then each matched on a control group based on gender, age and level of education. The AN control group (AN-C) included fifteen participants (14 female; 1 male). The ASD control group (ASD-C) included fifteen participants (13 male; 2 female).

### Self-report measures

The self-report measures described below were sent by post and participants were instructed to bring them completed on the day of their visit. The investigators checked the completeness of the questionnaires and got back to the participant when necessary so as to ensure that no items remained unanswered.

– *The Eating Attitudes Test-26 (EAT-26)*[22] was used to assess eating attitudes and behaviors. It includes three subscales: 1) Dieting (pathological avoidance of fattening foods and preoccupation with a thin body); 2) Bulimia and food preoccupations (bulimic tendencies such as bingeing and purging); 3) Oral control (perceived pressure to eat more and the degree self-control over eating).

– *The 13-item Beck Depression Inventory (BDI-13)*[23] was used to measure current dysphoric affects.

– *The Autism Spectrum Quotient (AQ)*[24] provides a total score and five subscores: Social skills, Attention switching, Attention to details, Communication skills and Imagination. Total scores are classified into one of four categories [25]: Typical (< 23;non ASD), Broader Autism Phenotype (BAP; 23–28), Medium Autism Phenotype (MAP; 29–34) or Narrow Autism Phenotype (NAP; > 35).

– *The Interpersonal Reactivity Index (IRI)*[26] is a multidimensional questionnaire on empathy which includes four subscales. Perspective taking (PT) evaluates attempts to take into consideration the points of view of others. Fantasy (FS) measures the propensity to identify with fictional characters. Personal distress (PD) assesses ‘self-oriented’ feelings and the tendency to feel anxious when confronted with negative situations. Empathic concern (EC) assesses “other-oriented” feelings of sympathy and concerns for unfortunate others.

– *The short forms of the Empathy Quotient (EQ-short) and Systemizing Quotient (SQ-short)*[27] were used to measure, respectively, empathy in vicarious situations and the individual’s drive to figure out the rules of a system or construct systems.

–For alexithymia, we used the *Bermond-Vorst Alexithymia Questionnaire-B (BVAQ-B)*[28]. It investigates five dimensions: verbalising emotional experiences (B1), daydreaming and fantasies (B2), identifying emotions (B3), proneness to being aroused by emotion-inducing events (B4), and analysing one’s own emotional states and reactions (B5). The BVAQ-B total score is obtained by the sum of all five subscales. The BVAQ-B Cognitive (BCOG) score corresponds to the sum of the scores on the Verbalising, Identifying, and Analysing subscales (B1 + B3 + B5, which is considered equivalent to the 20-item Toronto Alexithymia Scale score [TAS-20, [29]). The BVAQ-B Affective score (BAFF) corresponds to the sum of the scores on the Fantasising and Emotionalising subscales (B2 and B4, respectively). Low BVAQ-B Cognitive scores have been previously reported in ASD see [30] for a recent review.

### Data analyses

Statistical analyses were performed using R 2.11.1 [31]. For Body Mass Index (BMI) and each total scale score, the clinical groups were first compared to their respective control group using paired t-tests, and then ASD and AN were compared using Welch tests for independent samples. Standardized mean differences (SMD) (Cohen’s d, with pooled standard deviation) were used to estimate effect size on total scale as well as subscale scores.

A Principal Component Analysis (PCA) was used to analyse the correlation structure between AQ, IRI, and BVAQ-B subscales on the whole sample. PCA seeks to construct orthogonal linear combinations of all variables maximizing the amount of explained variance on scale scores, and it makes it possible to compute individual factor scores taking into account the relative contribution (loading) of each variable on the first principal components (PC). Since they can be interpreted geometrically as projections on the PCs, this in turn enables the study of subject proximities in a reduced-factor space. The number of components to retain was set at the first two PCs, to help visualize individual proximities in a 2D plane, but a scree plot was used to confirm that this was a reasonable choice. The contribution of each subscale to the factor structure can be summarized by a correlation circle where variables are represented as arrows starting from the centre of a unit circle. As long as variables remain far from the centre of this correlation circle, the angle formed by any two variables is proportional to their correlation, so that close variables are highly and positively correlated, while variables on opposite sides of the centre are negatively correlated, and variables whose angle is orthogonal are not correlated at all. Individual proximities can be assessed by Euclidean distances which are used as a similarity metric between subject coordinates in the factor plane.

## Results

**Figure 1:**
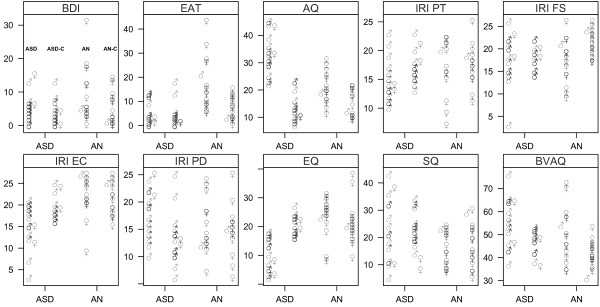
**Individual scores for ASD and AN patients and their matched.** Distribution of individual scores for males (♂) and females (♀) for ASD (on the left, in each panel) and AN patients (on the right). For each instrument, matched controls are shown right to their respective clinical group.

Mean scores (SD, range) on each test measure (all complete cases) are presented in Table 1. The distributions of individual scores for each group according to gender are displayed in Figure 1. Between-group comparisons for all total scale scores are shown in Table 2.

**Table 2:** Between-group comparisons for total scale scores

	**ASD vs ASD-C**		**AN vs AN-C**		**AN vs. ASD**		**Raw and std effect size (AN-ASD)**	
	**t**	**p**	**t**	**p**	**t**	**p**	**DM**	**ES**
BDI-13	1,29	0,217	2,21	**0,044**	1,39	0,179	3,33	0,507
BVAQ-B	2,82	**0,014**	2,28	**0,039**	−1,28	0,213	−5,27	−0,466
AQ	6,81	**0,000**	4,48	**0,001**	−5,28	**0,000**	−12,73	−1,927
IRI PT	−1,76	0,101	−0,11	0,916	1,38	0,179	2,20	0,505
IRI F	0,11	0,912	−3,19	**0,007**	−0,35	0,727	−0,67	−0,129
IRI EC	−2,49	**0,026**	0,43	0,671	3,64	**0,001**	6,53	1,330
IRI PD	2,82	**0,014**	0,34	0,742	−1,59	0,123	−2,73	−0,581
EQ-short	−5,59	**0,000**	0,80	0,438	5,66	**0,000**	12,93	2,068
SQ-short	0,39	0,701	−0,33	0,748	−2,14	**0,044**	−7,20	−0,781
EAT	1,04	0,317	3,03	**0,009**	3,71	**0,001**	11,60	1,353

Values in bold indicate significant differences.

### ASD vs ASD-C

The ASD participants and their controls (ASD-C) had similar Body Mass Index (BMI) (t = 0.70, p = 0.494, SMD = 0.24), BDI-13 and EAT-26 scores (Dieting: SMD = 0.25; Bulimia: SMD = 0.24; Oral control: SMD = 0.13). As expected, the ASD had higher AQ total and sub-scores (Social skills: SMD = 3.10; Attention switching: SMD = 2.31; Communication skills: SMD = 2.80; Imagination: SMD = 1.67), but with a lesser magnitude for AQ Attention to details (SMD = 0.74). The ASD had higher IRI PD and BVAQ-B scores (BCOG: SMD = 1.21; BAFF: SMD = 0.06), but lower EQ-short and IRI EC scores. Here, we found no significant group difference for the IRI PT, IRI FS and SQ-short scores.

### AN vs AN-C

As expected, the AN had significantly lower BMI (t = −6.46, p < 0.001, SMD = −4.64) and higher BDI-13, and EAT-26 total and sub-scores than their controls (Bulimia: SMD = 0.78; Oral control: SMD = 1.23; Dieting : SMD = 0.75). In addition, the AN had higher AQ total, Attention switching (SMD = 1.34) and Communication skills (SMD = 0.50) scores, but for the other AQ dimensions, the observed differences were moderate or small (Social skills: SMD = 0.52; Attention to details: SMD = 0.52; Imagination: SMD = 0.04). The AN also had higher BVAQ-B total and sub-scores (Affective BAFF: SMD = 0.85; Cognitive BCOG: SMD = 0.73). IRI scores were not significantly different, except for the IRI Fantasizing Scale scores which were lower among the AN than in their control group. The two groups were not significantly different on the EQ and SQ-short.

Using the AQ categories among the clinical participants, 10 AN and 1 ASD were classified as Typical (but this ASD participant reached a score of 15 on the ADOS), 2 ASD and 3 AN as BAP (1 restrictive AN and 2 binge/purging AN), 7 ASD and 2 AN as MAP (1 restrictive AN and 1 binge/purging AN), 5 ASD but no AN as NAP. Among the controls, all AN-C and 13 ASD-C were classified as Typical, and the 2 remaining ASD-C as BAP.

### AN vs ASD

As expected, compared to the ASD, the AN had significant lower BMI (t = 5.06, p < 0.001, SMD = 1.85) and higher EAT-26 total and sub-scores (Dieting SMD = 1.07; Bulimia SMD = 0.79; Oral control: SMD = 1.04), but did not differ on the BDI-13. The AN had significantly lower AQ total scores, which was accounted for by the fact the AN had lower scores for Social skills (SMD = −1.89), Attention switching (SMD = −1.12), Communication skills (SMD = −2.01) and Imagination (SMD = −1.09), but the two groups showed similar AQ Attention to details scores (SMD = −0.03). Finally, whereas the AN and ASD differed significantly for the IRI EC, EQ-short (both scores were higher among the AN group) and SQ-short scores (lower scores among the AN), the two groups had similar IRI PD, IRI PT, IRI FS and BVAQ-B total and sub-scores (Cognitive BCOG: SMD = −0.59; Affective BAFF: SMD = −0.03).

**Figure 2:**
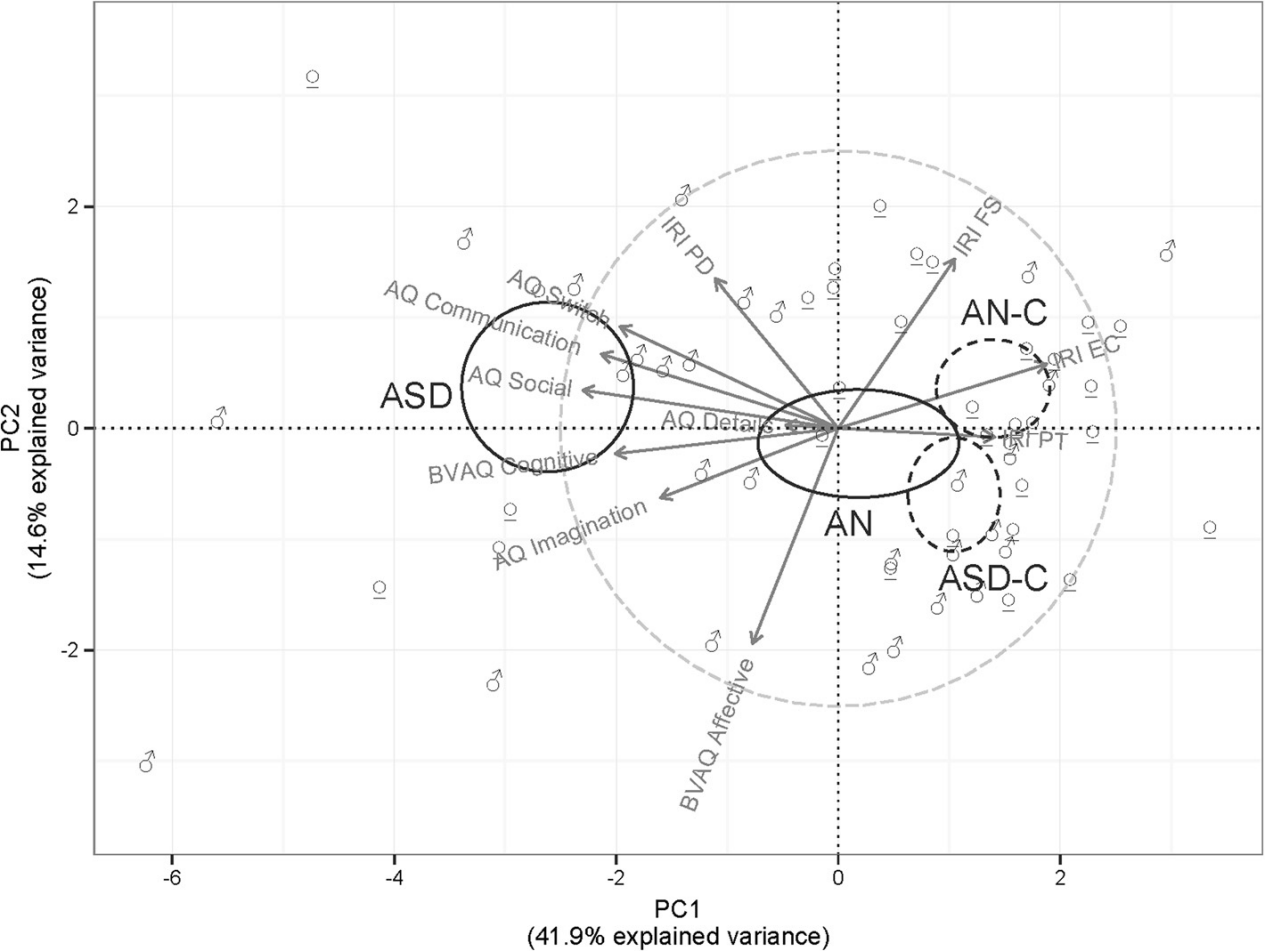
**Individuals factorial map.** Individual coordinates computed from the first two principal components of a PCA based on the correlation matrix of AQ, BVAQ-B and IRI scores. The correlation circle was superimposed on the factorial map of individual proximities.

Figure 2 shows individual coordinates computed from the first two principal components of a PCA based on the correlation matrix of AQ, BVAQ-B and IRI scores. These two PCs accounted for 56% of the total variance, and the relevance of this two-factor structure was confirmed by the presence of a clear knee in the plot of ordered eigenvalues (scree plot; available on request from the authors). The correlation circle was superimposed on the factorial map of individual proximities to facilitate factor interpretation. The first PC was largely dominated by all AQ subscale scores (except AQ Details), BVAQ-B Cognitive, and IRI EC and IRI PT scores, suggesting that these dimensions are strongly related. Most of the participants with high AQ and low IRI EC/PT scores are found on the left part of this factor space. The second dimension was mainly driven by the affective component of the BVAQ-B (BAFF) and a mix of IRI FS and IRI PD. This dimension might be thought of as reflecting the ability for daydreaming or identifying with fictional characters, and proneness to being aroused by emotion-inducing events.

The ellipse for patients (plain circle) and controls (dashed circle) was centered on average coordinates, or centroids. Although they rely on a Gaussian sampling distribution, they are helpful to visualize the typical location of ASD and AN patients and their controls. As expected, ASD patients were well separated from the rest of the participants, while AN patients were close to the centre of the factor space, with moderate levels on all trait measures.

With respect to gender (irrespective of group), males were found to exhibit high negative factor scores on the first PC (i.e., low empathy scores), while women were more likely to have high positive scores on the two PCs (i.e., high empathy scores and marked proneness to experiencing distress in emotional situations).

## Discussion

This study explored the level of cognitive and affective autistic traits in AN relative to both healthy controls and ASD patients. We found few arguments in favour of the suggestion that AN might be considered as a female variant of Asperger’s syndrome [32]. We observed between-group differences on some but not all the dimensions.

Regarding AQ categories, we found that a third of our AN sample shared certain endophenotypical markers of a broader/medium autism phenotype. In line with the conceptualization that autistic traits exist along a continuum [33], we used a dimensional approach and observed that both clinical groups reported a marked need for sameness: for the AQ Attention switching scores, the AN scored mid-way between the ASD and the controls. High scores on this AQ scale correspond to the neuropsychological performances that have been described in AN [9,10] and are in favour of considering cognitive remediation therapy for AN [34] as a promising mode of treatment. With respect to the other AQ scores, we observed clear difference between the two clinical groups. This pattern suggests no significant co-occurrence of any of the core features of ASD in AN.

In the study by Hambrook et al. on 22 AN and 45 controls, the AN had significantly higher AQ total and subscale scores for the AQ Social skills, Attention switching and Imagination scores [11]. The authors concluded this pattern of results suggests that patients with AN experience difficulties similar to those with ASD. In the present study, participants with AN were also found to have significantly higher AQ total and Attention switching scores, but the effect was smaller for AQ Communication and Social skills scores, and no differences were found for the AQ Attention to details and Imagination scores. Such discrepancies may be due to differences in sample size, and/or to variations in patients’ characteristics (i.e., greater age of illness onset and shorter illness duration in our AN sample). Here, by directly comparing AN and ASD patients, we were able to highlight that the difficulties are not as severe in both groups, and that the AN score midway between the ASD and the controls on some autistic traits.

Moreover, the AN and ASD groups differed on two important psychological dimensions within the Empathizing-Systemizing imbalance theory of autism (E-S theory: [35]). According to this model, individuals with ASD display poor Empathizing skills (inability to identify another person’s emotions and thoughts, and respond to these with an appropriate emotion) but high Systemizing skills (strong propensity to analyze, explore and construct a system, which provides a way of understanding and predicting non agent behaviours). Baron-Cohen et al. [36] argued that strong Systemizing and excellent attention to detail are related, and stated that the former relies on the latter. They further explained that strong systemizing might account for some of the non-social deficits found in autism, including repetitive behaviour, narrow repertoire of interests or resistance to change. Indeed, in order to systematically investigate the workings of a given phenomenon, it is best to only change its constituent features one at a time while keeping all else constant, and once a possible causal chain is identified, to verify the rule by repeating the sequence and observing the outcome [36], p.1378. Therefore, on the basis of this conceptualization and of the literature on the AN patients’ cognitive profile, we expected AN and ASD to have similar levels of Systemizing (SQ-short scores) in addition to attention to detail and need for sameness (AQ Details and Switching scores). However, our data seem to rule out the hypothesis that AN and ASD have the same Empathizing-Systemizing pattern.

Contrary to our prediction, but in line with the Hambrook et al. study [11], we found that adults with AN had similar Empathizing and Systemizing scores to their control group. In our study, the ASD group displayed low EQ-short but high SQ-short scores (the expected e-S pattern, typical of the ‘male brain type’), whereas the AN group reported a marked propensity to care for others and an ability to feel concerned in interpersonal situations (high EQ-short scores) but low Systemizing skills (an E-s pattern), which is considered the typical ‘female brain type’ in this model E-s pattern [27,35,37].

In the present psychometric study, in line with the idea that empathy is multidimensional, we included the IRI in addition to the EQ-short. The IRI includes two subscales that explore cognitive processes (Perspective Taking, Fantasy) and two measuring affective components (Personal Distress, Empathic Concern). Empathic Concern scores, which reflect the ability to experience appropriate emotions like compassion, tenderness or sadness for others, were significantly higher among the AN group than the ASD group. Nevertheless, we also observed that the AN group resembled the ASD group more than the controls on two other dimensions that are relevant for empathizing: the tendency to feel anxious when confronted to negative situations (example of an IRI Personal Distress item : “*Being in a tense emotional situation scares me*”) and the ability to identify with fictional characters (example of an IRI Fantasy item : “*When I watch a good movie, I can very easily put myself in the place of a leading character*”, negatively keyed). This is an important issue, as previous studies using experimental paradigms (e.g. facial or vocal expressions of emotions, pictures demonstrating basic emotions, film clips of social scenes, emotional melodies) revealed that individuals with AN have deficits in emotional awareness and recognition, and in understanding others’ emotional states [13]. As recently discussed [38-40], future studies are needed for better characterizing the pathways (e.g. maladaptive attachment, anhedonia, lack of embodiment) that may explain the relationships between interpersonal or social adjustment difficulties and disordered eating.

As early as the 1960s, Hilde Bruch highlighted the difficulties experienced by anorexic patients in perceiving or controlling their bodily sensations, and confusion in the identification of their mental states [41,42]. These deficits in interoception and emotional introspection in AN have since been explored further via studies of alexithymia, a multifaceted personality construct that has emerged in line with the idea that deficits in the ability to experience and symbolise emotions can have adverse effects on well-being [30]. Bagby and Taylor stated that alexithymic characteristics “*reflect deficits both in the cognitive-experiential domain of emotion response system and at the level of interpersonal regulation of emotion. [. . .] Lacking knowledge of their own emotional experiences, alexithymic individuals cannot readily imagine themselves in another person’s situation and are consequently unempathetic and ineffective in modulating the emotional states of others”*[43], p.30. Whereas alexithymia has been well documented in AN (see [13]), this is a current area of research in the autism literature. Interestingly, several recent experimental studies with ASD participants provided arguments for considering alexithymia as a potential part of the Broader Autism Phenotype. For instance, alexithymia has been identified in ASD family members [44,45], and in ASD alexithymia is associated with higher AQ scores, as well as with lower empathy scores, poorer facial expression decoding performances and decreased attention resource allocation to relevant facial areas [46-48]. Interestingly, the present study revealed no difference between the AN group and the ASD group on alexithymia scores (whereas the two clinical groups were significantly different from their respective control group), which might suggest that alexithymia accounts partly for impaired social skills in these two conditions. Yet, it appears that high alexithymia scores are not always associated with low empathy scores, as exemplified in the present sample of AN participants. This idea is further supported by the distribution of group scores across the first dimension of the PCA, where controls (AN-C and ASD-C) are located at one end of the axis and ASD at the other.

In order to provide a clearer picture of the potential overlap between socio-affective profiles in AN and ASD, and especially in order to determine whether there is a continuum between ASD, AN and healthy individuals, larger samples should be investigated. In particular, in order to account for discrepancies between our results and Hambrook et al. [11], future studies using larger cohorts of patients might be able to carefully take into account differences in AN patients’ characteristics (i.e., younger age of illness onset and longer illness duration in their AN sample) or AN subtype (2/3 of binge/purging subtype in our study but not specified in Hambrook et al. sample; see [38] for a recent discussion on this issue). Another potential limitation of the present study stems from the absence of IQ data collection in the AN group [49]. There is a need to address this issue in future studies. Moreover, our study relied on self-report measures only, so comparing AN and ASD behavioral performances on well-validated tasks as well as in terms of their neural activity while performing these tasks would be a useful next step (e.g. see [50]). In addition, the present study leaves an important issue unanswered: what could account for the fact AN and ASD are gender-related? Indeed, the female-to-male incidence ratio is opposite in AN (i.e. large majority of females) and ASD (i.e. large majority of males), as was the case in this data set. Gender differences in socio-affective skills have been well documented, and this argument underpins Baron-Cohen and colleagues’ ‘*extreme male brain*’ theory of autism. Emerging models for gender-biased neuropsychiatric disorders pointing to complex interactions between prenatal stress and gonadal hormones early in gestation [51], or the implication of testosterone and oxcytocin in human social and emotional behaviours [52,53], might prove to be very informative in furthering our understanding of the biological underpinnings of these gender differences. Although these issues were beyond the scope of the present project, it is interesting to note that in the present study we observed that for some measures, the AN resembled the male participants more (e.g. the ASD and the ASD-C alexithymia and IRI Fantasy scores), but also that participants with ASD resembled the female participants more on other dimensions (e.g. the AN and AN-C AQ Attention to details and IRI Personal Distress scores). Future studies, with a more balanced gender ratio across groups should be conducted to clarify this question.

## Conclusions

Overall, our study adds to the literature pointing to specific links between disturbed eating, emotional regulation and problems in social interactions. Nevertheless, as in the recent study by Pooni et al. [5], our results suggest that difficulties in AN are only superficially similar to those found in ASD. Our mixed results encourage the exploration of transdiagnostic similarities between these disorders, in particular in longitudinal studies which could help to determine whether or not the pattern observed here in AN is a mere effect of the acute phase of AN.

## Abbreviations

AN: Anorexia nervosa; ASD: Autism spectrum disorders; EOED: Early onset eating disorder; ADOS: Autism diagnostic observational schedule; ASD-C: ASD control group; AN-C: AN control group; EAT-26: The Eating attitudes test-26; BDI-13: The 13-item Beck depression inventory; AQ: Autism spectrum quotient; BAP: Broader autism phenotype; MAP: Medium autism phenotype; NAP: Narrow autism phenotype; IRI: The Interpersonal reactivity index; PT: Perspective taking; FS: Fantasy; PD: Personal distress; EC: Empathic concern; BVAQ-B: The Bermond Vorst alexithymia questionnaire- version B; TAS-20: The 20-item Toronto alexithymia scale; BAFF: BVAQ-B affective score; BCOG: BVAQ-B cognitive score; EQ-short: The short form of the Empathy quotient; SQ-short: The short form of the Systemizing quotient; BMI: Body mass index.

## Competing interests

The authors declare that they have no competing interest.

## Authors’ contributions

Conceived and designed the experiments: AC, DR, CV, J-L A, SB. Performed the experiments: AC, A-SM, DR, CV, CC, LP, FP, AP, SB. Analyzed the data: AC, A-SM, CL, CB, SB. Wrote the paper: AC, A-S M, CL, CC, SB. All authors read and approved the final manuscript.

## Pre-publication history

The pre-publication history for this paper can be accessed here:

http://www.biomedcentral.com/1471-244X/13/222/prepub
